# Evaluation of the Efficacy of the Addition of a Combination of Pyrimidine Nucleotides and Vitamin B1 and B12 to Standard Treatment in the Management of Painful Radiculopathy and in the Quality of Life of Patients

**DOI:** 10.3390/nu16234187

**Published:** 2024-12-04

**Authors:** Jordi Monfort, Irene Carrión-Barberà, Laura Tío, Javier Marante, Alicia López Vázquez, Teresa Bas, Lola Fernandez-Fuente-Burson, Miguel A. Caracuel, Antonio Oliveros-Cid, Virginia Gallart, Cintia Romera-López, José A. Román, David Abejón, Luis Javier Roca Ruíz, Alba Gurt, Fabiola Ojeda, Pedro Grima, Rebeca Aldonza

**Affiliations:** 1 Departament of Rheumatology, Hospital del Mar, 08003 Barcelona, Spain; jmonfort@psmar.cat (J.M.); mcarrion@psmar.cat (I.C.-B.); fojeda@psmar.cat (F.O.); 2 Hospital del Mar Research Institute, 08003 Barcelona, Spain; ltio@researchmar.net; 3 Hospital Universitario Jerez de la Frontera, 11407 Cádiz, Spain; javier.marante.sspa@juntadeandalucia.es; 4 Hospital de Orense, 32005 Ourense, Spain; alicia.lopez.vazquez@sergas.es; 5 Department of Traumatology and Orthopaedic Surgery, Hospital Universitario y Politécnico La Fe, 46026 Valencia, Spain; teresabas@gmail.com; 6 Hospital Quirónsalud Infanta Luisa, 41010 Sevilla, Spain; 7 Hospital Universitario Reina Sofía, 14004 Córdoba, Spain; caracuelr@gmail.com; 8 Hospital Viamed Montecanal, 50012 Zaragoza, Spain; aoliverosc.neuropolis@gmail.com; 9 Hospital Universitario Doctor Peset, 46017 Valencia, Spain; virgallart@hotmail.com; 10 Hospital General Universitario de Elche, 03203 Elche, Spain; cynthia.romera.lopez@hotmail.com; 11 Department of Rheumatology, Hospital Universitario y Politécnico La Fe, 46026 Valencia, Spain; roman_jan@gva.es; 12 Hospital Universitario Quirónsalud Madrid, 28223 Madrid, Spain; dabejongonzalez@gmail.com; 13 Hospital Universitario Virgen Macarena, 41009 Sevilla, Spain; luisjrocaruiz@gmail.com; 14 CAP Vila Olímpica, 08005 Barcelona, Spain; albagurt@gmail.com; 15 Affinity Petcare, 08902 Barcelona, Spain; pmgrima_oss@hotmail.com; 16 Ferrer Internacional, S.A., 08029 Barcelona, Spain

**Keywords:** radiculopathy, pain, quality of life, nucleotides, Vitamins B

## Abstract

**Background/Objectives**: Radiculopathy leads to pain, consequently reducing patient’s quality of life (QoL). Research indicates that certain nucleotides, such as cytidine and uridine, along with vitamins B1 and B12, may help alleviate pain and enhance QoL. This study assessed the impact of adding a supplement containing cytidine and uridine nucleotides and vitamins B1 and B12, alongside standard treatment, on radiculopathy-associated pain. **Methods**: A multicenter, prospective, two-cohort, randomized, open-label study was conducted. The control group received standard treatment, while the experimental group received standard treatment plus the supplement. The primary endpoint was pain reduction measured by a Visual Analog Scale (VAS). Secondary endpoints included functional improvement (Roland Morris questionnaire), clinical improvement (Clinical Global Impression [CGI] scale), and QoL improvement (EQ-5D-5L questionnaire). **Results**: A total of 122 patients were included from 17 centers across Spain. Both groups showed pain improvement, but the VAS reduction (control: 24.58 vs. experimental: 31.35) was not statistically significant. The Roland Morris score decreased significantly in the experimental group (estimate: −1.70, 95% CI −3.29 to −0.10; *p* = 0.038), and these patients were 5 times more likely to progress to a better CGI category (OR = 0.20, 95% CI 0.07 to 0.57; *p* = 0.003). No significant differences were observed in EQ-5D-5L scores or analgesic consumption. **Conclusions**: The addition of supplemental pyrimidine nucleotides and vitamins B1 and B12 to standard of care treatment improved radiculopathy functional and clinical outcomes. Regarding pain, however, although there was a numerical improvement, it did not reach statistical significance.

## 1. Introduction

Radiculopathy is a pathological process in which the spinal nerve roots are affected causing radicular pain that may occur along with other symptoms of decreased function. These radicular symptoms are not necessarily caused by mechanical compression, as it has been shown that they can also be produced by inflammatory reactions in adjacent structures, such as nerves or muscles [1]. Cervical radiculopathy has an annual incidence rate of 107.3 per 100,000 for men and 63.5 per 100,000 for women, whereas lumbar radiculopathy has a prevalence of approximately 3–5% of the population [2,3]. Additionally, up to two-thirds of adults experience neck or low back pain during their lifetime, representing a substantial clinical and socioeconomic burden [4]. Peripheral neuropathy, although distinct from radiculopathy, shares similarities in that it involves injury to sensory or motor nerves, but it occurs in the peripheral nervous system rather than at the nerve roots [5].

Schwann cells are glial cells responsible for producing the myelin sheaths that surround the nerves, which are essential for the conduction of nerve signals. The pathophysiology of radiculopathies includes demyelination caused by lesions affecting Schwann cells. Therefore, regenerating these cells after a nerve injury is critical to maintain the myelin sheaths and address the neuropathy [6]. Successful repair processes in damaged nerves associated with neuropathies include, among others, axonal growth and myelination [5]. Treatment with uridine has been shown to reduce apoptosis and oxidative stress in experimental models of sciatic nerve injury, suggesting its potential in peripheral nerve regeneration by promoting anti-apoptotic and antioxidant effects [7].

Cytidine and uridine are critical pyrimidine nucleotides that contribute to nerve regeneration by supporting the synthesis of phospholipids and glycolipids, key components of the myelin sheath and other nerve structures. Their participation stimulates metabolic activity, facilitating the regeneration of the myelin sheath and the restoration of nerve conduction and muscle trophism [8]. Additionally, cytidine and uridine play key roles in mRNA synthesis, including the production of tubulin mRNA, which is critical for supporting axonal growth and regeneration [9,10]. Following nerve injury, elevated nucleotide levels become essential to sustain RNA synthesis, addressing the increased demand for pyrimidine nucleotides required for efficient nerve repair [5]. Studies have demonstrated that pyrimidine nucleotides administration enhances myelination and nerve conduction in both in vitro and in vivo models, including significant improvements in axonal and myelin thickness following nerve injury. For example, in rats with sciatic nerve injury, daily intramuscular administration of uridine monophosphate (UMP) and cytidine monophosphate (CMP) over 40 days resulted in improved conduction velocity of afferent fibers. This treatment also enhanced both myelin area and thickness. By day 60, significant improvements were observed in fiber and axon areas, as well as in myelin thickness, compared to untreated controls [11]. Furthermore, the addition of UTP and CMP to human nerve cell cultures stimulated cell division and the synthesis of proteins and lipids, processes essential for nerve fiber repair. Treated cells exhibited a significant increase in galactocerebroside, the primary component of myelin, compared to untreated cells. Additionally, other key myelin components, including sphingomyelin, phosphatidylcholine, and phosphatidylethanolamine, also showed increases, although these were not statistically significant [12]. Therefore, exogenous pyrimidine nucleotides are associated with increased cell growth rate, protein and lipid synthesis, thus favoring nerve repair.

In addition to pyrimidine nucleotides, vitamins B1 (thiamine) and B12 (cobalamin) play essential roles in nerve repair and regeneration, further supporting the restoration of myelin and neuronal function. These vitamins are essential for protecting nerves from environmental damage and supporting the regeneration process following injury. Vitamin B1 acts as an antioxidant, reducing oxidative stress and facilitating energy metabolism, while vitamin B12 promotes the maintenance and repair of myelin sheaths, supporting neuronal survival and enhancing remyelination [13]. Their importance in nerve metabolism is well-established, with deficiencies potentially leading to myelin sheath disintegration and axonal damage in both the central and peripheral nervous systems [14]. In cases of peripheral nerve injury, the presence of these vitamins supports the development of new cell structures and promotes regeneration [13]. Notably, vitamin B12 has shown promise in accelerating nerve regeneration processes compared to other B vitamins, particularly by increasing myelin sheath thickness at 30- and 45-days post-injury [15]. However, it is important to note that excessive levels of vitamin B6 can have neurotoxic effects, potentially leading to peripheral neuropathy [16]. Therefore, without adequate levels of these vitamins, there is an increased risk of permanent nerve degeneration, pain, and the development of peripheral neuropathy [13].

Pyrimidine nucleotides and B vitamins play dual roles in both neural regeneration and pain modulation. Purinergic P2 are G-protein-coupled receptors such as P2Y1 and P2Y2, are activated by purine and pyrimidine nucleotides, influencing nociception by lowering the activation threshold of transient receptor potential (TRP) channels [17]. Specifically, uridine triphosphate (UTP) and uridine diphosphate (UDP) interact with P2Y receptors in neurons and glial cells, exhibiting potential analgesic effects by inhibiting spinal pain transmission. These purinergic receptors regulate intricate signaling pathways and molecular mechanisms within the nervous system, contributing to the maintenance of physiological health and mitigating disease progression. In fact, P2 receptor activation has shown potential analgesic effects, reducing pain intensity in various conditions, such as diabetic neuropathy and trauma-related injuries [18,19]. Activation of P2Y2 receptors in astrocytes also promotes cell proliferation and migration [20]. This receptor-mediated response includes upregulation of genes associated with neurotrophins, neuropeptides, growth factors, extracellular matrix proteins, and fibronectin, all of which play crucial roles in neuroprotection and repair [21]. Furthermore, the combined administration of uridine and cytidine has demonstrated significant efficacy in alleviating pain intensity across various conditions, including diabetic neuropathy, lumbar and cervical pain, as well as trauma-induced compressive injuries [8,22]. Moreover, animal studies also suggest that combining certain B vitamins, such as B1 and B12, may improve neuropathies, enhance motor control, and alleviate both nociceptive and neuropathic pain [8,23].

Given their analgesic properties, the combination of nucleotides and vitamin B12 has been evaluated in patients experiencing pain associated with peripheral neuropathies, compressive neuralgias, low back pain, and carpal tunnel syndrome. In these studies, pain intensity was measured using aVAS, a tool in which patients rate their pain from 0 (no pain) to 10 (worst pain imaginable) [24,25,26,27]. The results show that this combination is effective in reducing pain and, therefore, may reduce the need for other drugs that have undesirable adverse effects which can worsen patient’s QoL. Furthermore, the combination of nucleotides with vitamin B12 is suitable for long-term use, since it has a relatively low profile of adverse effects, representing an alternative to other commonly used analgesics, such as non-steroidal anti-inflammatory drugs (NSAIDs), corticosteroids, or opioids whose effectiveness and safety remain uncertain [28,29].

The purpose of this prospective, randomized study was to assess the response to treatment with a combination of uridine, cytidine, vitamin B12, and vitamin B1, in addition to standard of care treatment, in patients with painful radiculopathy at any location.

## 2. Materials and Methods

### 2.1. Study Design and Study Population

This study was a randomized, open-label, prospective, comparative, interventional study with two cohorts: an experimental group, which received standard treatment plus a combination of pyrimidine nucleotides and B12 and B1 vitamins, and a control group, which received only standard treatment.

Patients in the control group received standard treatment for painful radiculopathy prescribed by their doctors, following standard clinical practice. Concomitant medications categorized by ATC (Anatomical Therapeutic Chemical) Level, as well as baseline medication used in both groups, are summarized in Supplementary Tables S1 and S2. All patients in the experimental treatment group received a standardized dose of the food supplement, consisting of a daily tablet of food supplement containing 300 mg of uridine (pyrimidine nucleotide), 100 mg of cytidine (nucleotide), 2640 µg of thiamine nitrate (vitamin B12) and 1493 mg of cyanocobalamin (vitamin B1) in addition to the usual treatment. The supplement dosages were adjusted in accordance with the recommended daily nucleotide intake specified by the Spanish Agency for Food Safety and Nutrition (AESAN) in its 2012 report (AESAN-2012-008) [30]. The tablet was not to be crushed, but swallowed whole, accompanied by any liquid, and preferably taken before meals. This supplement was not commercially available in Spain and was provided by the sponsor.

Subjects were recruited over 29 months, with each participant attending three visits during the study: before treatment, 4 ± 1 weeks after treatment initiation, and at 8 ± 1 weeks after treatment initiation.

Participant patients were randomized in a 1:1 ratio through the electronic case report form according to a randomization list prepared by Clinscience Spain by means of a custom function programmed in R statistical software [31] and stratified for opiates.

Participants in the study were of both genders, aged 18 to 85 years with painful radiculopathy of any etiology at spinal level confirmed through imaging techniques during the last 12 months. They also had to have upper neuropathic pain, in a pain VAS ≥ 60 mm and pain evolution of less than 6 months. In patients with relapsing disease, the absence of a previous episode in the last 6 months was needed. All subjects provided written informed consent before any study procedure was performed.

Patients diagnosed with painful radiculopathy who were candidates for surgical treatment or with any active bleeding or severe liver and/or kidney failure or acute asthma or rhinitis or dyshematopoiesis or who were known to be intolerant to any of the components of the food supplement were excluded.

### 2.2. Endpoints

The primary endpoint of the study was to assess the degree of pain improvement. Secondary objectives included evaluating the functional improvement of the limbs affected (movement, muscle strength and other neurological symptoms), the impact and improvement of the patients’ QoL and the patient’s medication intake for painful radiculopathy throughout the study according to the improvement or worsening of pain.

The VAS score, a vertical line of 100 mm with extreme expressions of a symptom at each end (0 representing the lowest and 100 the highest intensity), was used to measure the absolute change from baseline in pain intensity as described by the patient. To assess the degree of physical disability caused by painful radiculopathy, the Roland Morris’s scale [32] and Clinical Global Impression (CGI) scale were performed [33,34]. The Roland Morris’s scale consists of 24 yes/no questions regarding daily activities, with one point added for each positive response, and 0 points for each negative response. The worst possible result is 24/24, and the best is 0/24 [32]. The CGI scale includes two subscales: one assessing symptom severity (CGI-S) and another evaluating the improvement due to treatment interventions (CGI-I). CGI-S was used at baseline and CGI-I scale at post-baseline visits. Both subscales use an 8-point Likert scale: for CGI-S, from 0 (not assessed) to 7 (extremely sick), and for CGI-I, from 0 (not assessed) to 7 (much worse).

To assess patients’ QoL, the EQ-5D-5L questionnaire was used [35]. Patients assessed their health status, first by rating severity across five dimensions: mobility, self-care, daily life activities, pain/discomfort, and anxiety/depression (descriptive system) and then through a VAS for a more general assessment, both at baseline and final visits. Additionally, patients were asked to record changes in medication in a diary, noting the active ingredient, frequency, and dose. For those participants who accepted to wear a Fitbit activity band, daily walked distance and sleep quality (total time of deep sleep, total time of light sleep, total time of being awake after having slept at night) were recorded.

### 2.3. Statistical Methods

The protocol planned various statistical methods: descriptive analyses were conducted for categorical variables to determine absolute and relative frequencies, while for continuous variables, metrics such as mean, standard deviation, median, minimum, and maximum were calculated. When using the VAS for pain, descriptive statistics were applied at baseline and subsequent visits, alongside a Mixed Model for Repeated Measures (MMRM) to compare pre- and post-experimental period pain assessments. Results from the Roland Morris’s questionnaire and the CGI were analyzed using descriptive tables, mean calculation for the scores of Roland Morris’s questionnaire, and MMRM. For QoL evaluation with the EQ-5D-5L questionnaire, frequency tables for each dimension and mean values on the associated VAS were reported. Physical activity and sleep quality were assessed using data from activity bands, including descriptive statistics and MMRM comparisons. Estimates of treatment effects, including differences in mean scores between groups, were derived from the MMRM model with corresponding two-sided 95% confidence intervals (CI).

Statistical tests were also conducted to assess potential differences in baseline characteristics. Missing data were handled according to ICH-E9 and CPMP/EWP/1776899 Rev.1 guidelines [36]. For the primary endpoint, only available data were used, and missing data were not imputed. For continuous variables, MMRM were robust to missing data when missing at random (MAR). The Available Data Only (ADO) approach was used for all other variables, and participants with missing post-baseline data were excluded from the analysis for the respective variable.

No previous studies have used such high nucleotide doses. Assuming a mean difference of 10 mm in the VAS scale and a standard deviation of 17 mm based on prior studies [37], a sample size of 112 patients was initially determined, accounting for a 15% drop-out rate and a significance level of 5%. Additionally, an interim analysis was planned at 50–70% data availability to recalculate the sample size, executed when 58.9% of initially planned patients were recruited, and the result of the recalculation was 126 patients. In the end, 127 patients were screened.

The analysis was performed with R statistical software [31], version 4.3.0, through the integrated development environment [38], version 2023.03.1 “Cherry Blossom”, with a significance level of *p* < 0.05 for all tests.

## 3. Results

### 3.1. Patient Disposition and Baseline Characteristics

**Figure 1:**
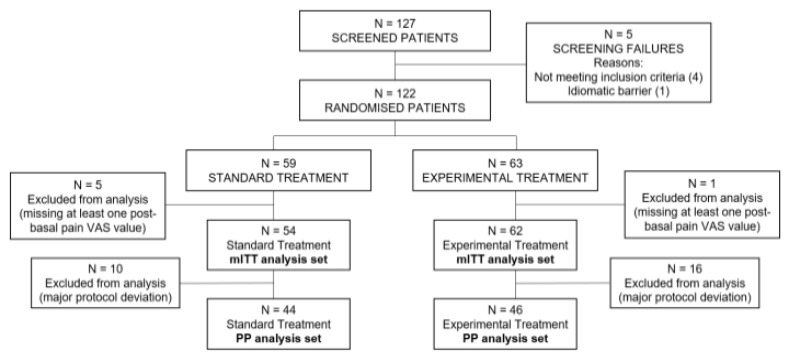
Patient disposition diagram. VAS: pain visual analog scale, PP: per protocol, mITT: modified intent-to-treat.

Of the 127 patients screened, 122 met eligibility criteria and were randomized into both arms. Fifty-nine patients were assigned to the control group, and 63 patients to the experimental treatment group. For efficacy assessment, two analysis populations were determined; All randomized patients with a baseline value for the primary endpoint and at least one follow-up value constituted the modified intent-to-treat (mITT) group (116 patients), on which the primary analysis for efficacy endpoints was performed. Additionally, patients who met the selection criteria and had no major protocol deviations, comprised the per protocol (PP) group (90 patients), which was included only in a sensitive analysis for the primary endpoint to control the effects of protocol non-compliance (Figure 1).

Demographic and baseline characteristics of the subjects are provided in Table 1. Statistical tests confirmed that there were no significant differences between groups (*p* > 0.05) at baseline in gender, race, age, BMI, pain according to the VAS, total scores on the Roland Morris’ scale, or initial responses to the EQ-5D-5L questionnaire, indicating that both groups were comparable. Although there was no safety evaluation in this study, it should be noted that there were no study discontinuations due to safety issues.

### 3.2. Primary Endpoint: Pain Reduction (VAS Score)

**Figure 2:**
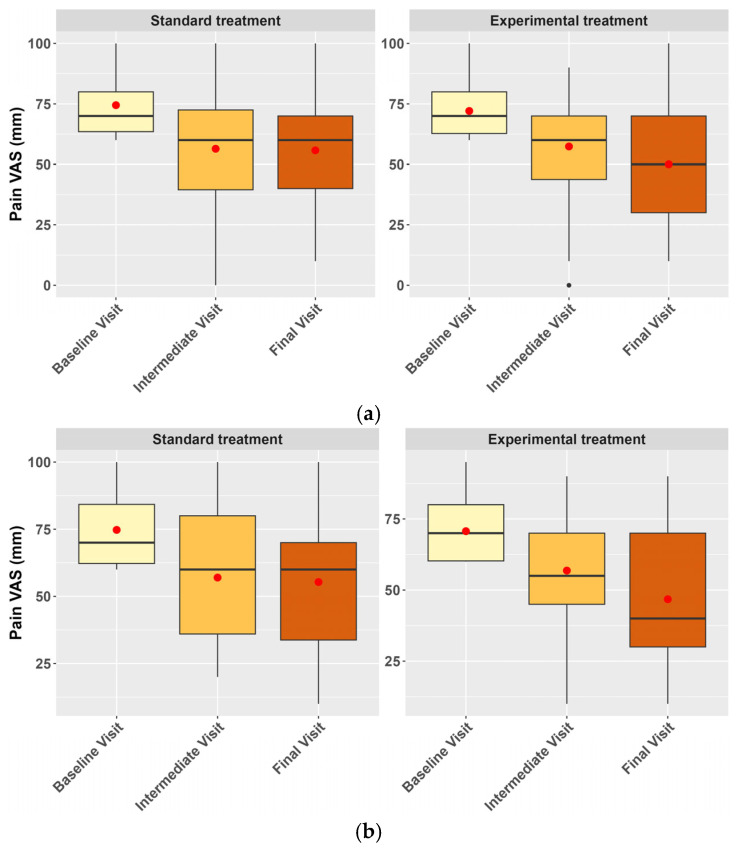
Boxplots representing the evolution of the pain VAS score across the study visits by treatment group in the mITT population analysis set (**a**) and the PP population analysis set (**b**).

In both mITT and PP populations, an improvement in pain (measured as the mean change in the VAS score) over the course of the study was observed for both study groups (Figure 2). However, no statistically significant differences were found between the control and the experimental groups (estimate: 3.12, CI 95% −4.43–10.68, *p* = 0.416 in mITT population; estimate: 3.90, confidence interval (CI) 95% −4.54–12.34, *p* = 0.363 in PP population set). Nevertheless, differences emerged when setting a stratification factor for opioid consumption, as patients using opioids reported higher pain levels than those not consuming them, regardless of the treatment assigned and the visit number (estimate: 5.21, CI 95% 0.51–9.90, *p* = 0.030 in mITT population; estimate: 5.97, CI 95% 0.63–11.32, *p* = 0.029 in PP population set).

### 3.3. Secondary Endpoint: Functional and Clinical Improvement

**Figure 3:**
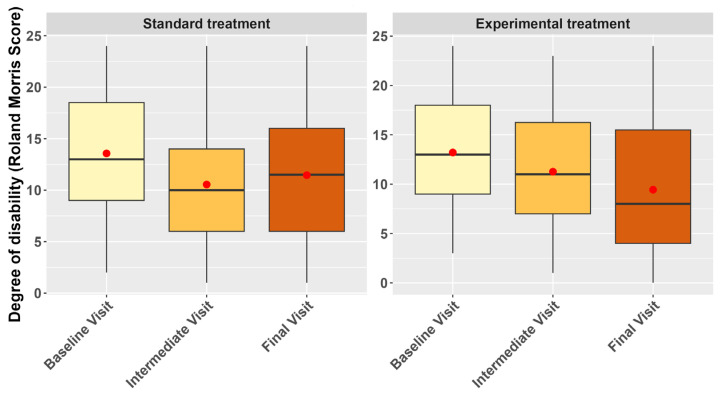
Boxplots representing the evolution of the degree of disability (Roland Morris Score) across the study visits by treatment group in the mITT population analysis set.

Patients’ functional and clinical improvement was assessed using the results from the Roland Morris’ questionnaire and the CGI scale. Figure 3 shows the evolution of the degree of disability, which resulted in a significant decrease over time, with the estimate for the intermediate visit being −2.74 (95% CI = −3.90 to −1.57, *p* < 0.001). Similarly, the estimate for the final visit was −2.09 (95% CI = −3.25 to −0.93, *p* < 0.001).

Even though there was no statistically significant effect of the experimental treatment on the outcome when not considering the time of the visit, the interaction term between the experimental treatment group and the final visit was significant with a decrease of 1.70 units (CI 95%: −3.29 to −0.10, *p* = 0.038), in the score during the final visit for those in the experimental treatment group compared to those in the control group.

**Figure 4:**
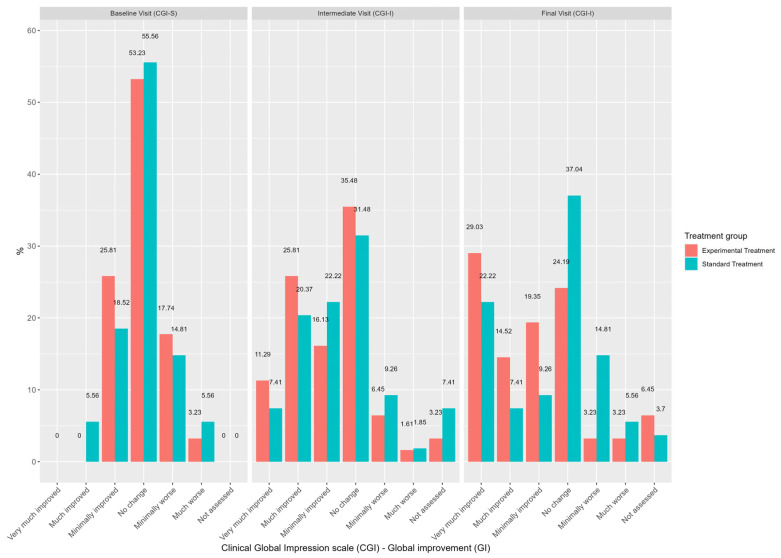
Bar chart representing the evolution of the Clinical Global Impression scale (CGI) across the study visits by treatment group in the mITT analysis population set.

The CGI scale measured the severity of the symptoms at post-baseline visits resulting from treatment intervention (Figure 4). Displacement analysis, performed using the proportional odds model, showed that patients in the experimental treatment group were five times more likely to evolve to a better CGI category at the final visit than those in the standard treatment group (OR = 0.20, CI 95% = 0.07 to 0.57, *p* = 0.003).

### 3.4. Quality of Life (EQ-5D-5L)

Changes in QoL of patients were assessed through the EQ-5D-5L questionnaire. Results of each EQ-5D-5L dimension by treatment group and visit (baseline or final) are presented in Table 2. Although a significant change in each section’s score of the questionnaire was observed over the course of the study for both groups, neither the experimental treatment nor the opiate treatment were found to influence the outcomes.

For a more general assessment, patients also assessed their health status using a VAS. The EQ-VAS results indicated improvement over time in both groups, with baseline EQ-VAS values influencing scores across study visits. Patients with lower baseline EQ-VAS values showed greater QoL improvement. However, the experimental treatment did not show a significant impact on the EQ-VAS scores compared with the control treatment.

### 3.5. Medication Intake for Pain Management

Regarding patient’s medication intake for painful radiculopathy, analgesics were the most prescribed category for both treatment groups with paracetamol (acetaminophen) being the most used medication, followed by pregabalin and tramadol. Concomitant medications categorized by ATC Level, as well as baseline medication used in both groups, are summarized in Supplementary Tables S1 and S2. After analyzing the change from baseline in the total sum of prescribed pain medications, the experimental treatment did not demonstrate a significant impact (estimate: −0.02; 95% CI = −0.51 to 0.48; *p* = 0.946). However, a notable increase of 0.66 units in the total sum of prescribed pain medications was observed in both groups at the final visit compared to baseline (95% CI = 0.16 to 1.16; *p* = 0.011). Additionally, patients who used opioids in both groups were prescribed 0.46 units more pain medications (95% CI = 0.09 to 1.03; *p* = 0.016).

### 3.6. Exploratory Endpoints: Physical Activity

Patient’s physical activity was also assessed as an exploratory objective. The experimental treatment only affected walking distance when considering the interaction between visit number and treatment: patients in the experimental treatment group exhibited a small decrease in the walked distance over time, although this was not clinically relevant. However, sleep quality did not appear to be significantly affected by any treatment.

## 4. Discussion

We report a comparative study of the use of a combination of pyrimidine nucleotides with vitamins B12 and B1 plus standard treatment vs. standard treatment alone in the management of painful radiculopathy, using a pain VAS score as the primary outcome measure. The primary endpoint analysis was performed in two populations (mITT and PP) and different covariates were established.

Previous studies have shown that nucleotides and vitamin B1 and B12 combinations reduce pain and improve patient’s care and QoL in other painful conditions [38]. For example, the NUBES study, a 60-day randomized, double blind, controlled trial, reported encouraging results, as treatment with nucleotides (cytidine and uridine) in addition to vitamin B12 vs. B-complex vitamins was found to significantly reduce low back pain intensity in a VAS after 30 days of treatment however, the reduction was equivalent by day 60 [27]. Similarly, in our study, we observed that neither of the treatment groups studied showed statistically significant differences in the pain VAS score vs. baseline, although the visit number (intermediate or final) did show an effect. Nevertheless, it is worth noting that the pain VAS score showed a numerical decline in patients who received the experimental supplement. This lack of significance could be explained by an important limitation in the study, the low power achieved to detect the differences in the mean change from baseline to the final visit in the pain VAS, due to the small sample size and a greater variability than initially expected. Thus, potential differences between groups might have been detected with a larger sample size. Additionally, as defined by the International Association for the Study of Pain (IASP), pain is a subjective experience that can be influenced by different degrees of biological, psychological, and social factors [39]. Therefore, even though pain has to be evaluated during patient’s clinical assessments, functional improvement may be considered clinically more relevant, as it provides a measure of how the condition is affecting patient’s daily activities.

In this regard, we also included the assessment of patients’ functional and clinical improvement, as well as the impact on QoL, as secondary objectives. The Roland Morris’s questionnaire showed that time (measured as visit number) is a predictor of patients’ functional improvement, since the disability score decreased significantly over time in the control and experimental groups. However, patients in the experimental treatment group showed a greater functional improvement in the final visit, with a significant decrease of 1.70 units in the degree of disability score, compared to patients in the control group. 

Similarly, although the visit number was found to be a significant predictor of the improvement on the severity of the symptoms in the CGI scale for both groups, patients in the experimental group were five times more likely to evolve to a better CGI category at the final visit than those in the control group. This analysis also revealed an additional significant predictor on patients’ progress, as patients with worse basal CGI values were less likely to worsen in the CGI category. The final visit represented a significant change over the course of the study for both study groups, indicating that the visit number is another important factor influencing the scores of each section of the questionnaire. However, the experimental treatment was not found to influence the outcomes. Similar results were obtained in the EQ-VAS, where a worse baseline value tended to show higher improvements in QoL. The final visit was a predictor of an increase in the EQ-VAS score compared to the baseline visit, indicating an improvement over time in both study groups. In addition, the experimental treatment did not show a significant impact on the EQ-VAS scores compared with the control treatment.

On the other hand, when analyzing the established covariates, our study showed that consuming opioids was associated with an increase in the pain VAS score, regardless of the treatment received or visit number. This result could be explained by a worse initial condition in those patients who were prescribed opioids than the ones not consuming them, or even to a selection bias, since doctors tend to prescribe opioids when they, or the patient, feel that the condition is worse. Dose and potency of analgesic treatments were not considered in the analysis, nor were pain type and its evolution; therefore, it would be valuable to include such analyses in future studies.

Previous research has demonstrated that nucleotides in combination with B12 vitamin are safe and well tolerated [26]. However, while our study showed that the total amount of prescribed pain medications significantly increased for both groups, there were no significant differences between treatment groups. Finally, patient’s physical activity was assessed as an exploratory objective, but the differences found were not clinically relevant, as the experimental treatment only affected walking distance when considering the interaction between visit number and treatment, with patients in the experimental treatment group exhibiting a small decrease in the walked distance over time.

## 5. Conclusions

In conclusion, adding the studied combination of nucleotides and vitamins B1 and B12 to standard treatment (e.g., anti-inflammatories, corticosteroids, or opioids) provides a benefit in the improvement of functional and clinical symptoms in patients with painful radiculopathy, with the advantage of a relatively low adverse event profile. However, although numerically directional, no statistically significant differences were observed on the degree of pain improvement, as measured by the VAS. A future study with a larger sample size would be advisable, as an important limitation of the study was the low power to detect differences between groups.

## Figures and Tables

**Table 1:** Demographic and baseline characteristics of the mITT population set.

Demographics	Experimental Group (n = 62) n (%)	Control Group (n = 54) n (%)	Total (n = 116) n (%)
Gender, n (% *)			
Male	31 (50%)	30 (55.56%)	61 (52.59%)
Female	31 (50%)	24 (44.44%)	55 (47.41%)
Race, n (% *)			
Caucasian	61 (98.39%)	50 (92.59%)	111 (95.69%)
Asian	0 (0.00%)	1 (1.85%)	1 (0.86%)
Other	1 (1.61%)	3 (5.56%)	4 (3.45%)
**Age**			
Mean (SD)	50.74 (14.04)	53.17 (12.86)	51.87 (13.5)
Median (P25, P75)	49.5 (42, 60)	56 (43, 62)	53 (42, 62)
**Height**			
Mean (SD)	169.68 (11.14)	167.85 (11.18)	168.83 (11.15)
Median (P25, P75)	171 (160, 178)	167 (160.25, 178)	170 (160, 178)
**Weight**			
Mean (SD)	81.35 (17.26)	81.35 (17.26)	79.47 (17.01)
Median (P25, P75)	80.5 (65.45, 93.5)	75.2 (65.25, 85.22)	78 (65.07, 92)
**BMI**			
Mean (SD)	28.21 (5.25)	27.35 (4.72)	27.81 (5.01)
Median (P25, P75)	27.84 (24.76, 30.81)	26.75 (24.38, 29.84)	27.04 (24.54, 30.65)
**Time since diagnosis (days)**			
Mean (SD)	239.92 (671.03)	324.65 (944.14)	279.36 (807.13)
Median (P25, P75)	76 (21, 139.75)	81.5 (21.5, 140)	77.5 (21, 140.25)
Opiate treatment, n (% *)			
Yes	28 (45.16%)	23 (42.59%)	51 (43.97%)
No	34 (54.84%)	31 (57.41%)	65 (56.03%)
Difficulty or pain in mobility, n (% *)			
Yes	56 (90.32%)	47 (87.04%)	103 (88.79%)
No	6 (9.68%)	6 (11.11%)	12 (10.34%)
Not assessed	0 (0.00%)	1 (1.85%)	1 (0.86%)
Spontaneous pain, n (% *)			
Yes	47 (75.81%)	35 (64.81%)	82 (70.69%)
No	14 (22.58%)	17 (31.48%)	31 (26.72%)
Not assessed	1 (1.61%)	1 (1.85%)	2 (1.72%)
Not available	0 (0.00%)	1 (1.85%)	1 (0.86%)
Referred pain, n (% *)			
Yes	60 (96.77%)	49 (90.74%)	109 (93.97%)
No	2 (3.23%)	4 (7.41%)	6 (5.17%)
Not assessed	0 (0.00%)	1 (1.85%)	1 (0.86%)
Peripheral neurological involvement, n (% *)			
Yes	39 (62.9%)	31 (57.41%)	70 (60.34%)
No	22 (35.48%)	22 (40.74%)	44 (37.93%)
Not assessed	1 (1.61%)	1 (1.85%)	2 (1.72%)
Sensory neurological involvement, n (% *)			
Yes	30 (48.39%)	29 (53.7%)	59 (50.86%)
No	32 (51.61%)	23 (42.59%)	55 (47.41%)
Not assessed	0 (0.00%)	2 (3.7%)	2 (1.72%)
Motor neurological involvement, n (% *)			
Yes	14 (22.58%)	12 (22.22%)	26 (22.41%)
No	48 (77.42%)	40 (74.07%)	88 (75.86%)
Not assessed	0 (0.00%)	2 (3.7%)	2 (1.72%)
Presence of joint reflexes, n (% *)			
Yes	53 (85.48%)	43 (79.63%)	96 (82.76%)
No	8 (12.9%)	9 (16.67%)	17 (14.66%)
Not assessed	1 (1.61%)	2 (3.7%)	3 (2.59%)
Presence of skin sensitivity, n (% *)			
Yes	40 (64.52%)	32 (59.26%)	72 (62.07%)
No	22 (35.48%)	21 (38.89%)	43 (37.07%)
Not assessed	0 (0.00%)	1 (1.85%)	1 (0.86%)
Trophic disorders, n (%*)			
Yes	0 (0.00%)	1 (1.85%)	1 (0.86%)
No	61 (98.39%)	52 (96.3%)	113 (97.41%)
Not assessed	0 (0.00%)	1 (1.85%)	1 (0.86%)
Not available	1 (1.61%)	0 (0.00%)	1 (0.86%)
Impaired muscle strength, n (% *)			
Yes	12 (19.35%)	9 (16.67%)	21 (18.1%)
No	50 (80.65%)	44 (81.48%)	94 (81.03%)
Not assessed	0 (0.00%)	1 (1.85%)	1 (0.86%)
CGI-S, n (% *)			
Borderline ill	0 (0.00%)	3 (5.56%)	3 (2.59%)
Mildly ill	16 (25.81%)	10 (18.52%)	26 (22.41%)
Moderately ill	33 (53.23%)	30 (55.56%)	63 (54.31%)
Markedly ill	11 (17.74%)	8 (14.81%)	19 (16.38%)
Severely ill	2 (3.23%)	3 (5.56%)	5 (4.31%)

* Note: The denominator for the percentages is the number of subjects in each group/column. SD: standard deviation, BMI: body mass index.

**Table 2:** EQ-5D-5L test by treatment and by visit—mITT population set.

EQ-5D-5L Test	Day	Scale	Control Treatment n (%)	Experimental Treatment n (%)
**Mobility**	Baseline Visit	I have no problems in walking about (1)	10 (18.52%)	13 (20.97%)
		I have slight problems in walking about (2)	19 (35.19%)	21 (33.87%)
		I have moderate problems in walking about (3)	17 (31.48%)	17 (27.42%)
		I have severe problems in walking about (4)	7 (12.96%)	11 (17.74%)
		I am unable to walk about (5)	1 (1.85%)	0 (0.00%)
		Mode	2	2
	Final Visit	I have no problems in walking about (1)	17 (31.48%)	24 (38.71%)
		I have slight problems in walking about (2)	17 (31.48%)	15 (24.19%)
		I have moderate problems in walking about (3)	15 (27.78%)	13 (20.97%)
		I have severe problems in walking about (4)	4 (7.41%)	7 (11.29%)
		No answer	1 (1.85%)	3 (4.84%)
		Mode	2	1
**Self-care**	Baseline Visit	I have no problems washing or dressing myself (1)	19 (35.19%)	23 (37.1%)
		I have slight problems washing or dressing myself (2)	15 (27.78%)	16 (25.81%)
		I have moderate problems washing or dressing myself (3)	16 (29.63%)	19 (30.65%)
		I have severe problems washing or dressing myself (4)	2 (3.7%)	4 (6.45%)
		I am unable to wash or dress myself (5)	2 (3.7%)	0 (0.00%)
		Mode	1	1
	Final Visit	I have no problems washing or dressing myself (1)	21 (38.89%)	32 (51.61%)
		I have slight problems washing or dressing myself (2)	14 (25.93%)	16 (25.81%)
		I have moderate problems washing or dressing myself (3)	16 (29.63%)	6 (9.68%)
		I have severe problems washing or dressing myself (4)	1 (1.85%)	5 (8.06%)
		I am unable to wash or dress myself (5)	1 (1.85%)	0 (0.00%)
		No answer	1 (1.85%)	3 (4.84%)
		Mode	1	1
**Usual activities**	Baseline Visit	I have no problems doing my usual activities (1)	7 (12.96%)	4 (6.45%)
		I have slight problems doing my usual activities (2)	13 (24.07%)	16 (25.81%)
		I have moderate problems doing my usual activities (3)	22 (40.74%)	26 (41.94%)
		I have severe problems doing my usual activities (4)	8 (14.81%)	14 (22.58%)
		I am unable to do my usual activities (5)	4 (7.41%)	1 (1.61%)
		No answer	0 (0.00%)	1 (1.61%)
		Mode	3	3
	Final Visit	I have no problems doing my usual activities (1)	10 (18.52%)	12 (19.35%)
		I have slight problems doing my usual activities (2)	19 (35.19%)	26 (41.94%)
		I have moderate problems doing my usual activities (3)	18 (33.33%)	10 (16.13%)
		I have severe problems doing my usual activities (4)	2 (3.7%)	8 (12.9%)
		I am unable to do my usual activities (5)	4 (7.41%)	3 (4.84%)
		No answer	1 (1.85%)	3 (4.84%)
		Mode	2	2
**Pain/discomfort**	Baseline Visit	I have no pain or discomfort (1)	1 (1.85%)	0 (0.00%)
		I have slight pain or discomfort (2)	8 (14.81%)	12 (19.35%)
		I have moderate pain or discomfort (3)	18 (33.33%)	25 (40.32%)
		I have severe pain or discomfort (4)	21 (38.89%)	23 (37.1%)
		I have extreme pain or discomfort (5)	6 (11.11%)	2 (3.23%)
		Mode	4	3
	Final Visit	I have no pain or discomfort (1)	4 (7.41%)	7 (11.29%)
		I have slight pain or discomfort (2)	15 (27.78%)	21 (33.87%)
		I have moderate pain or discomfort (3)	18 (33.33%)	16 (25.81%)
		I have severe pain or discomfort (4)	9 (16.67%)	14 (22.58%)
		I have extreme pain or discomfort (5)	6 (11.11%)	1 (1.61%)
		No answer	2 (3.7%)	3 (4.84%)
		Mode	3	2
**Anxiety/depression**	Baseline Visit	I am not anxious or depressed (1)	17 (31.48%)	23 (37.1%)
		I am slightly anxious or depressed (2)	11 (20.37%)	17 (27.42%)
		I am moderately anxious or depressed (3)	14 (25.93%)	9 (14.52%)
		I am severely anxious or depressed (4)	10 (18.52%)	7 (11.29%)
		I am extremely anxious or depressed (5)	2 (3.7%)	6 (9.68%)
		Mode	1	1
	Final Visit	I am not anxious or depressed (1)	20 (37.04%)	27 (43.55%)
		I am slightly anxious or depressed (2)	14 (25.93%)	14 (22.58%)
		I am moderately anxious or depressed (3)	11 (20.37%)	10 (16.13%)
		I am severely anxious or depressed (4)	2 (3.7%)	6 (9.68%)
		I am extremely anxious or depressed (5)	5 (9.26%)	1 (1.61%)
		No answer	2 (3.7%)	4 (6.45%)
		Mode	1	1
		**Total**	54 (100%)	62 (100%)

## Data Availability

The datasets presented in this article are not publicly available. They are the property of Ferrer Internacional, S.A. and are available from the corresponding author upon reasonable request, subject to privacy and ethical considerations.

## Supplementary Materials

The following supporting information can be downloaded at: https://www.mdpi.com/article/10.3390/nu16234187/s1, Table S1: Concomitant medication by ATC (Anatomical Therapeutic Chemical) Level at baseline visit—mITT population set; Table S2: Medication at baseline visit—mITT population set.
