# Supplementary material for: Evaluation of the Efficacy of the Addition of a Combination of Pyrimidine Nucleotides and Vitamin B1 and B12 to Standard Treatment in the Management of Painful Radiculopathy and in the Quality of Life of Patients
Source: Nutrients. 2024 Dec 4;16(23):4187. doi: 10.3390/nu16234187 (PMC11644542; doi:10.3390/nu16234187)
Supplement: Supplementary file 1 [file nutrients-16-04187-s001.zip › nutrients-3300128-supplementary.pdf]

SUPPLEMENTARY MATERIAL

Supplementary Table S1. Concomitant medication by ATC (Anatomical Therapeutic Chemical) Level at baseline visit – mITT population set

ATC Level 2	ATC Level 4	Standard treatment N (%*)	Experimental treatment N (%*)	All N (%*)
**Agents acting on the renin-angiotensin system**		0 (0.00 %)	1 (0.67 %)	1 (0.33)
	Angiotensin ii receptor blockers (ARBS), plain	0 (0.00 %)	1 (0.67 %)	1 (0.33)
**Analgesics**		87 (58 %)	104 (69.8 %)	191 (63.88)
	Anilides	25 (16.67 %)	33 (22.15 %)	58 (19.4)
	Natural opium alkaloids	2 (1.33 %)	2 (1.34 %)	4 (1.34)
	Other analgesics and antipyretics	31 (20.67 %)	33 (22.15 %)	64 (21.4)
	Other opioids	19 (12.67 %)	23 (15.44 %)	42 (14.05)
	Phenylpiperidine derivatives	1 (0.67 %)	2 (1.34 %)	3 (1)
	Pyrazolones	8 (5.33 %)	11 (7.38 %)	19 (6.35)
	Salicylic acid and derivatives	1 (0.67 %)	0 (0.00 %)	1 (0.33)
**Antianemic preparations**		4 (2.67 %)	0 (0.00 %)	4 (1.34)
	Folic acid and derivatives	1 (0.67 %)	0 (0.00 %)	1 (0.33)
**Antiinflammatory and antirheumatic products**		31 (20.67 %)	24 (16.11 %)	55 (18.39)
	Acetic acid derivatives and related substances	3 (2 %)	3 (2.01 %)	6 (2.01)
	Coxibs	5 (3.33 %)	5 (3.36 %)	10 (3.34)
	Other antiinflammatory and antirheumatic agents, non-steroids	0 (0.00 %)	1 (0.67 %)	1 (0.33)
	Oxicams	0 (0.00 %)	2 (1.34 %)	2 (0.67)
	Propionic acid derivatives	23 (15.33 %)	13 (8.72 %)	36 (12.04)
**Calcium channel blockers**		0 (0.00 %)	1 (0.67 %)	1 (0.33)
	Dihydropyridine derivatives	0 (0.00 %)	1 (0.67 %)	1 (0.33)
**Corticosteroids for systemic use**		5 (3.33 %)	4 (2.68 %)	9 (3.01)
	Glucocorticoids	5 (3.33 %)	4 (2.68 %)	9 (3.01)
**Drugs for acid related disorders**		1 (0.67 %)	0 (0.00 %)	1 (0.33)
	Proton pump inhibitors	1 (0.67 %)	0 (0.00 %)	1 (0.33)
**Drugs for functional gastrointestinal disorders**		1 (0.67 %)	0 (0.00 %)	1 (0.33)
	Propulsives	1 (0.67 %)	0 (0.00 %)	1 (0.33)
**Muscle relaxants**		9 (6 %)	6 (4.03 %)	15 (5.02)
	Other centrally acting agents	9 (6 %)	6 (4.03 %)	15 (5.02)
**Other nervous system drugs**		2 (1.33 %)	1 (0.67 %)	3 (1)
	Drugs used in opioid dependence	1 (0.67 %)	1 (0.67 %)	2 (0.67)
	Other parasympathomimetics	1 (0.67 %)	0 (0.00 %)	1 (0.33)
**Psycholeptics**		1 (0.67 %)	1 (0.67 %)	2 (0.67)
	Benzodiazepine derivatives	1 (0.67 %)	1 (0.67 %)	2 (0.67)
**Vitamins**		5 (3.33 %)	0 (0.00 %)	5 (1.67)
	Vitamin B1 in combination with vitamin B6 and/or vitamin B12	3 (2 %)	0 (0.00 %)	3 (1)
	Vitamin D and analogues	2 (1.33 %)	0 (0.00 %)	2 (0.67)
	Vitamin B12 (cyanocobalamin and analogues)	3 (2 %)	0 (0.00 %)	3 (1)
**Total**		150 (50.17)	149 (49.83)	299 (100)

Note: *The denominator for the percentages is the number of total prescribed medications.

Supplementary Table S2. Medication at baseline visit – mITT population set

Preferred Term	Experimental treatment N (%*)	Standard treatment N (%*)
Aceclofenac	1 (1.61 %)	0 (0 %)
Acetylsalicylic acid	0 (0 %)	1 (1.85 %)
Amitriptyline	3 (4.84 %)	4 (7.41 %)
Amlodipine	1 (1.61 %)	0 (0 %)
Calcifediol	0 (0 %)	2 (3.7 %)
Celecoxib	2 (3.23 %)	2 (3.7 %)
Chondroitin sulfate	1 (1.61 %)	0 (0 %)
Codeine	1 (1.61 %)	1 (1.85 %)
Cyanocobalamin	0 (0 %)	2 (3.7 %)
Cyclobenzaprine	0 (0 %)	1 (1.85 %)
Dexamethasone	1 (1.61 %)	5 (9.26 %)
Dexamethasone acetate	1 (1.61 %)	0 (0 %)
Dexketoprofen	5 (8.06 %)	8 (14.81 %)
Diazepam	4 (6.45 %)	5 (9.26 %)
Diclofenac	0 (0 %)	2 (3.7 %)
Diclofenac sodium	1 (1.61 %)	0 (0 %)
Duloxetine	1 (1.61 %)	0 (0 %)
Etoricoxib	3 (4.84 %)	3 (5.56 %)
Fentanyl	2 (3.23 %)	1 (1.85 %)
Folic acid	0 (0 %)	1 (1.85 %)
Gabapentin	7 (11.29 %)	8 (14.81 %)
Hydroxocobalamin; pyridoxine hydrochloride; thiamine hydrochloride	0 (0 %)	3 (5.56 %)
Ibuprofen	1 (1.61 %)	2 (3.7 %)
Indometacin	1 (1.61 %)	0 (0 %)
Ketoprofen	1 (1.61 %)	0 (0 %)
Ketorolac	0 (0 %)	1 (1.85 %)
Lorazepam	1 (1.61 %)	0 (0 %)
Lornoxicam	2 (3.23 %)	0 (0 %)
Medazepam	0 (0 %)	1 (1.85 %)
Metamizole	11 (17.74 %)	7 (12.96 %)
Metamizole magnesium	0 (0 %)	1 (1.85 %)
Metoclopramide	0 (0 %)	1 (1.85 %)
Naloxone	1 (1.61 %)	1 (1.85 %)
Naproxen	5 (8.06 %)	12 (22.22 %)
Naproxen sodium	1 (1.61 %)	1 (1.85 %)
Omeprazole	0 (0 %)	1 (1.85 %)
Oxycodone	1 (1.61 %)	1 (1.85 %)
Paracetamol	33 (53.23 %)	25 (46.3 %)
Pilocarpine hydrochloride	0 (0 %)	1 (1.85 %)
Prednisone	2 (3.23 %)	0 (0 %)
Pregabalin	22 (35.48 %)	19 (35.19 %)
Tapentadol	3 (4.84 %)	4 (7.41 %)
Tapentadol hydrochloride	2 (3.23 %)	0 (0 %)
Telmisartan	1 (1.61 %)	0 (0 %)
Tizanidine	2 (3.23 %)	3 (5.56 %)
Tramadol	18 (29.03 %)	15 (27.78 %)
Vitamin B12 NOS	0 (0 %)	1 (1.85 %)
Patients with no medication prescribed	7 (11.29 %)	4 (7.41 %)
**Total**	**62 (100%)**	**54 (100%)**

Note: * The denominator for the percentages is the number of total patients in that group.
